# Dynamic pathway analysis of genes associated with blood pressure using whole genome sequence data

**DOI:** 10.1186/1753-6561-8-S1-S106

**Published:** 2014-06-17

**Authors:** Pingzhao Hu, Andrew D Paterson

**Affiliations:** 1 The Centre for Applied Genomics, The Hospital for Sick Children, 686 Bay Street, Toronto, ON, M5G 0A4, Canada; 2 Program in Genetics and Genome Biology, The Hospital for Sick Children, 686 Bay Street, Toronto, ON, M5G 0A4, Canada; 3 Dalla Lana School of Public Health, University of Toronto, Health Sciences Building, 155 College St, Toronto, ON, M5T 3M7, Canada; 4 Department of Biochemistry and Medical Genetics and George and Fay Yee Centre for Healthcare Innovation, University of Manitoba,745 Bannatyne Avenue, Winnipeg, MB, R3E 0W3, Canada

## Abstract

Groups of genes assigned to a pathway, also called a module, have similar functions. Finding such modules, and the topology of the changes of the modules over time, is a fundamental problem in understanding the mechanisms of complex diseases. Here we investigated an approach that categorized variants into rare or common and used a hierarchical model to jointly estimate the group effects of the variants in a pathway for identifying enriched pathways over time using whole genome sequencing data and blood pressure data. Our results suggest that the method can identify potentially biologically meaningful genes in modules associated with blood pressure over time.

## Background

It has long been recognized that genetic analysis of longitudinal phenotypic data is important for understanding the genetic architecture and biological variations of complex diseases. The analysis can help identify the stage of disease development at which specific genetic variants play a role. However, the statistical methods to analyze longitudinal genetic data are limited. A commonly used approach is to analyze the longitudinal genetic traits by averaging multiple response measurements obtained at different time points from the same individual. This approach may miss a lot of useful information related to the variability of repeated genetic traits, although it is simple and computationally less expensive. Linear mixed models have also been used for repeated measures data [1].

Recently, there has been a shift to testing rare variants, mostly using next-generation sequence technologies, for association with complex diseases. We explored dynamic pathway-based analysis of genes associated with blood pressure over time using whole genome sequencing data. We first performed gene-based association analysis at each of the 3 time points by stratifying the variants into rare and common. Then we performed pathway enrichment analysis separately at each time point. Finally, we built pathway crosstalk network maps using the enriched pathways to identify potential subnetworks associated with blood pressure over time.

## Methods

### Data description

For genotype data, we analyzed sequencing data of the 142 unrelated individuals on chromosome 3, which includes 1,215,120 variants. For phenotype data, we analyzed the simulated phenotypes of replicate 1. We analyzed 2 quantitative traits: systolic blood pressure (SBP) and Q1. SBP was measured at 3 time points (T1, T2, and T3), and was close to normally distributed (data not shown) after treatment effect adjustment (see below). There are 31 functional loci (genes) on chromosome 3 that influence the simulated SBP. Q1 was simulated as a normally distributed phenotype but not influenced by any of the genotyped single-nucleotide polymorphisms. It also has no correlation with SBP measured at T1, T2, and T3. The Pearson correlation of SBP at the 3 time points with Q1 based on the 142 unrelated individuals is −0.09 (*p *value = 0.27), −0.02 (*p *value = 0.78), and −0.006 (*p *value = 0.94), respectively. Q1 was generated primarily to facilitate assessment of type 1 error.

### Adjust treatment effects

It has been shown that the association between measured blood pressure and underlying genotype is potentially confounded by antihypertensive treatment [2]. Following Cui et al [3], we adjusted SBP of subjects receiving antihypertensive medications by adding a constant value of 10 mm Hg at the 3 study exams (n = 22, 51, and 73; 15.5%, 35.9%, and 51.4%, respectively). Such a strategy had higher power than the alternatives [2].

### Analyze common and rare genetic variants using hierarchical models

We applied an extended hierarchical generalized linear model [4] to simultaneously analyze rare and common variants at the gene level. The model can be summarized as follows: Assume that the observed values of a given quantitative trait (SBP or Q1) are denoted as $y=\left(y_{1},\cdots \;,y_{n}\right)$ and the predictor variables, that are variants, can be categorized into 2 groups: rare (minor allele frequency <1%) and common variants (minor allele frequency ≥1%). The number of variants in the rare and common groups are $J_{1}$ and $J_{2},$ respectively. The extended hierarchical generalized linear model to fit the rare and common variants in a given gene can be expressed as a multiplicative form for the linear predictor *η *of individual *i*:

$$\eta_{i}=\beta_{0}+\overset{2}{\underset{k=1}{\sum }}g_{k}\overset{J_{k}}{\underset{j\in G_{k}}{\sum }}\beta_{j}Z_{ij}$$

where $z_{ij}$ is the predictor of main-effect for individual *i *at genetic variant *j *in group $G_{k},$ equaling to the number of minor alleles for an additive coding and $Z_{i}=\left(z_{i1},\ldots ,z_{iJ}\right),$ where $J=\overset{2}{\underset{k=1}{\sum }}J_{k}$ is the total number of variants. $g_{k}$ represents the group effect for $J_{k}$ variants in group $G_{k}$. *β *is a vector of all the coefficients and the intercept *β*.

The mean of *y *is related to the linear predictor η via a link function *h*:

$$E\left(y_{i}|Z_{i}\right)=h^{-1}\left(Z_{i}\beta \right)$$

The data distribution is expressed as $\rho \left(y|Z\beta ,\theta \right)=\overset{n}{\underset{i=1}{\prod }}\rho \left(y_{i}|Z_{i}\beta ,\theta \right),$ where θ is a dispersion parameter, and the distribution $\rho \left(y_{i}|Z_{i}\beta ,\theta \right)$ takes normal distribution. Because there are many highly correlated variants in a given gene in next-generation sequencing studies, a hierarchical framework is constructed for priors of the distributions of coefficients (*g *and *β*) in the model. The method was implemented in R package BhGLM (http://www.ssg.uab.edu/bhglm/).

We assigned the genetic variants to a gene if they were in the gene or within 10 kilobases (kb) of either side of the gene. We performed 2 analyses to evaluate the association between genotype and SBP at each study exam separately. First, we divided the variants within a gene into rare (*k *= 1) and common variants (*k *= 2). Separately we analyzed all the genetic variants in a gene, irrespective of allele frequency. Our main objective was to estimate gene effects $g_{k}$ and to test the hypothesis $g_{k}=0,$*k *= 1 (rare variants) and *k *= 2 (common variants) for the first analysis and *k *= 1 (rare and common variants) for the second analysis. We corrected for multiple testing using the Benjamini and Hochberg method [5].

### Dynamic pathway analysis

We mapped the approximately 1200 genes on chromosome 3 to the c2 curated pathways (version 3) from the Broad Institute (http://www.broadinstitute.org/gsea/msigdb/), which includes 2934 gene sets collected from 186 Kyoto Encyclopedia of Genes and Genomes (http://www.genome.jp/kegg/), 430 Reactome, 217 BioCarta pathways, 880 canonical pathways, 825 biological process, and 396 molecular function gene ontology terms. We kept only the pathways with at least 5 genes in our data set, which left 531 pathways for analysis.

There are different ways to test for genes associated with an excess of SBP in the same pathway. We used the "gene set enrichment test" implemented in the limma R package [6]. The approach uses the Wilcoxon signed rank test to compute a *p *value to test the hypothesis that a given gene set tends to be more highly ranked than would be expected by chance. The ranking is based on a *t*-like test statistic, and here we used the *z *statistics from the hierarchical model described in above Section (Analyze common and rare genetic variants using hierarchical models). The test is essentially a streamlined version of the gene set enrichment analysis approach introduced by Subramanian et al [7].

We performed dynamic pathway crosstalk analysis between each pair of time points using the enriched pathways with a nominal *p *value of <0.05. Two pathways were considered to crosstalk if they shared at least 1 functional locus (gene). This ensures that each of pathway and its crosstalk has biological meaningfulness. We built pathway crosstalk subnetworks using Cytoscape (http://www.cytoscape.org/).

## Results

Given a false discovery rate (FDR) of 0.05 at the gene-level analysis, we identified 116, 57, 2, and 0 significant genes for SBP measured at T1, T2, T3, and Q1, respectively, using rare variants. However, there were no significant genes for SBP measured at the 3 time points and Q1 using common variants. Of those significant genes from the rare variant analysis, 4, 1, 0, and 0 were true positives (Table 1) for SBP measured at T1, T2, T3, and Q1, respectively. We observed that these 4 genes had significant positive associations with SBP (Table 1). For the gene-level analysis irrespective of allele frequency we identified many significant genes (468, 415, 306, and 214 for SBP measured at T1, T2, T3, and Q1, respectively) (Table 2). However, the vast majority of them were false positives (see false-positive rate analysis below), implying that, irrespective of allele frequency, the analysis strategy had a grossly inflated type I error, possibly as a result of linkage disequilibrium between variants. We calculated the false-positive rate and false-negative rare for T1, T2, T3, and Q1 using the 2 analysis approaches. We defined the positive as those genes that had an adjusted *p *value smaller than 0.05, and the negative as those genes that had an adjusted *p *value larger than or equal to 0.05. As shown in Table 2, irrespective of allele frequency, the analysis approach had many false positives compared with the approach that stratified by allele frequency. The genes based on common variants used in the first analysis had no power to detect the genetic association between genotypes and SBP.

**Table 1:** Significant association of causal genes with rare variants with SBP at T1 and T2 based on chromosome 3 gene-based tests

Time period and gene		Rare variants				Common variants			
			
		Estimate	SE	*z *Value	Adjusted *p *value	Estimate	SE	*z *Value	Adjusted *p *value
T1	*ABTB1*	0.71	0.24	3.01	0.0321	−0.15	0.21	−0.70	0.99
	*SCAP*	0.87	0.22	4.00	0.0058	−0.02	0.08	−0.27	0.99
	*PROK2*	3.30	0.90	3.66	0.011	−0.19	0.21	−0.93	0.99
	*MUC13*	0.65	0.22	2.96	0.035	0.00	0.08	0.02	1.0
T2	*SCAP*	1.06	0.27	3.89	0.0076	−0.08	0.08	−1.00	0.95

Note: There were no significant associations for causal genes including only either rare or common variants at T3 (or for Q1).

**Table 2:** False-positive rate (FPR) and false-negative rate (FNR) of gene-based analyses

Time period or trait	Gene-based stratified by allele frequency				Gene based	
		
	Rare variants		Common variants		Rare and common variants	
			
	FPR (%)	FNR (%)	FPR (%)	FNR (%)	FPR (%)	FNR (%)
T1	9.6	86.7	0.0	100.0	39.2	53.3
T2	4.8	96.7	0.0	100.0	34.9	63.3
T3	0.2	100.0	0.0	100.0	25.6	66.7
Q1	0.0	100.0	0.0	100.0	17.8	76.7

To further evaluate the whole-spectrum of power of the approaches to identify causal genes, we drew the receiver operating characteristic curves for each type of strategy at each time point and estimated its area under the curve. We found that each analysis strategy has different power to identify causal genes at different time points. Overall, the analysis based on rare variants had the largest power at T3, which also had larger power to detect disease-causing genes than common variants at T2.

Using the *z *statistic obtained from modeling rare variants in a gene, we did not find significant pathways associated with SBP at FDR of 0.05 level based on gene set enrichment analysis. However, given a nominal *p *value cutoff of 0.05, we identified the same 3 enriched pathways for SBP measured at 3 time points but not for Q1 (Table 3). Each of these 3 pathways included 1 "functional" gene (*FLNB*), which had 286 rare variants with 1 functional variant (chr3: 58109162, explained 0.00273 of the variance for SBP).

**Table 3:** Enriched pathways found in T1, T2, and T3

Pathway names	No. of genes	No. of genes on chr. 3	No. of functional loci	*p *Value of T1	*p *Value of T2	*p *Value of T3
Actin filament-based process	114	10	1	0.021	0.016	0.018
Actin cytoskeleton organization and biogenesis	104	10	1	0.021	0.016	0.018
Cytoskeleton organization and biogenesis	205	12	1	0.030	0.013	0.022

**Figure 1:**
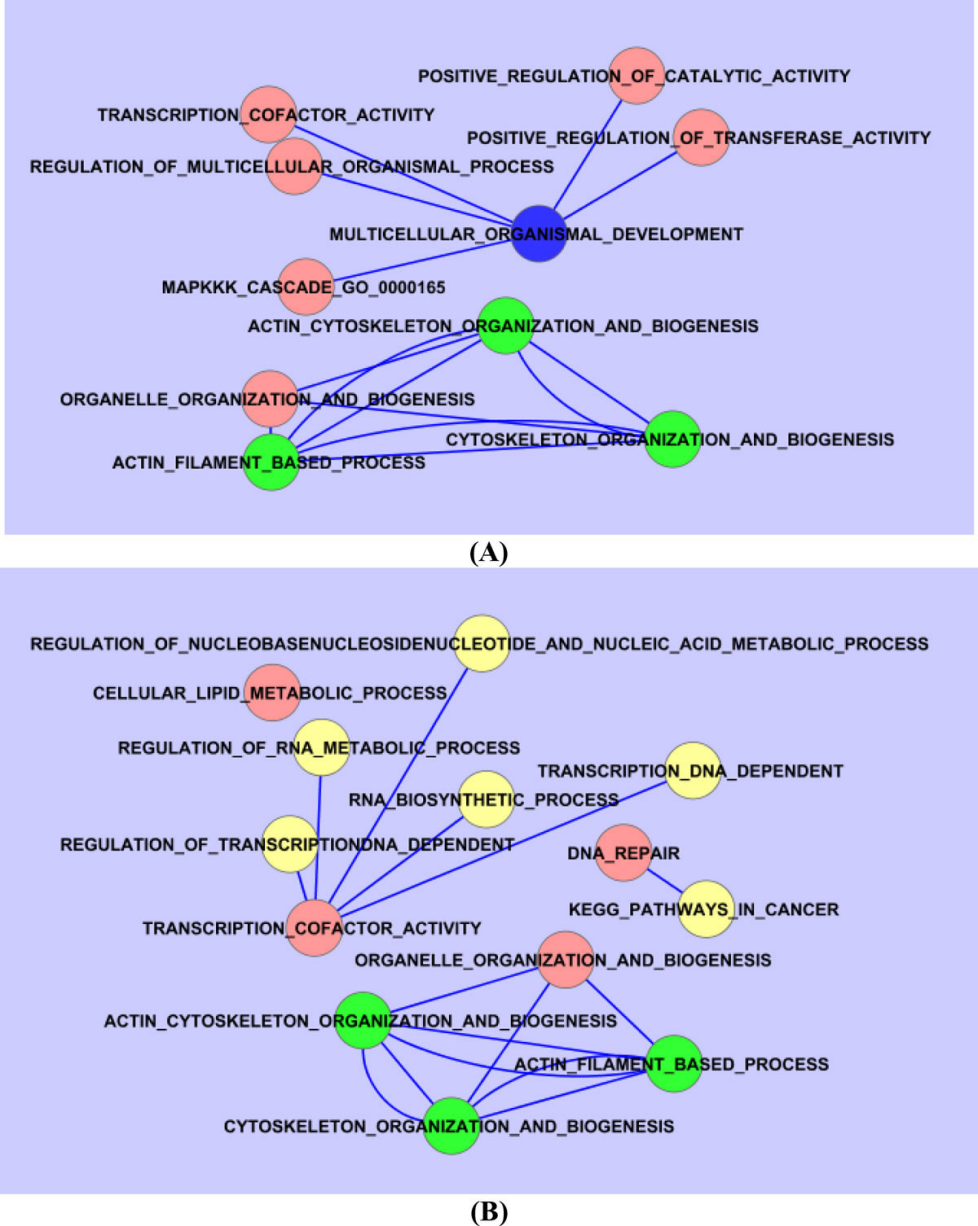
**Dynamic pathway crosstalk: (A) T1 and T2; (B) T2 and T3**. Pathways with blue, red and yellow are enriched at T1, T2, and T3, respectively. Pathways with green are enriched at both T1 and T2 (A) and at both T2 and T3 (B). A single line between 2 pathways indicates that each of the 2 pathways is enriched in only 1 of the 2 time points; a double-line between 2 pathways indicates that both pathways are enriched in both T1 and T2 (A) and in both T2 and T3 (B).

To identify pathway crosstalk, we built 2 pathway subnetworks (Figure 1) for the pathways with nominal *p *value smaller than 0.05. Two pathways crosstalk if at 2 time points they included at least 1 common true gene. As shown in Figure 1, we found that there was a subnetwork formed by the "actin filament based process," the "actin cytoskeleton organization and biogenesis," and the "cytoskeleton organization and biogenesis and organelle organization and biogenesis" pathways that were consistently enriched across adjacent time periods (T1 → T2 and T2 → T3).

## Discussion

In this study, we evaluated the associations between rare and common genetic variants and the simulated quantitative trait (SBP) measured at 3 time points at the gene and pathway levels. We found that joint modeling all the variants (rare and common) together had a high type I error, which may be a result of linkage disequilibrium between common and rare variants, or the average effect between rare and common variants. However, a strategy that categorized variants into rare or common and used a hierarchical model to jointly estimate the group effects showed rare variants had higher power to detect functional loci than did common variants. Although we did not find statistically significant pathways associated with SBP (FDR of the 0.05 level), we showed some enriched pathways shared across time at a nominal *p *value cutoff of 0.05. Interestingly, we also found a subnetwork with 3 enriched pathways that showed crosstalk between each pair of time points, suggesting the dynamic pathway crosstalk may have a key role in the pathogenesis of SBP. It should be noted the "'functional" loci defined in simulation answers provided by Genetic Analysis Workshop 18 (GAW18) organizers were polymorphic based on all individuals, but they may be not polymorphic in the unrelated individuals analyzed in this study. In this case, some functional loci (or genes) may not have effects in the unrelated data, which may lead to the bias in calculation of false negatives.

## Conclusions

In summary, we proposed a framework to identify dynamic pathways which have the potential in regulating SBP via analyzing repeated traits with next-generating sequencing. This can generate insights into the progressive mechanisms of the underlying disease. This analysis strategy can also be applied to examine the mechanisms that drive the progression of complex diseases.

## Competing interests

The authors declare that they have no competing interests.

## Authors' contributions

PH designed the study, performed the data analysis, and drafted the manuscript. ADP participated in designing the study. ADP supervised the study. All authors helped revise the manuscript. All authors read and approved the final manuscript.
